# Southern Tick-Associated Rash Illness: Florida's Lyme Disease Variant

**DOI:** 10.7759/cureus.15306

**Published:** 2021-05-28

**Authors:** Ramy Abdelmaseih, Bilal Ashraf, Randa Abdelmasih, Sandi Dunn, Hesham Nasser

**Affiliations:** 1 Internal Medicine, University of Central Florida College of Medicine, Ocala, USA

**Keywords:** stari, lyme disease, pancytopenia, tick-bite, zoonotic disease

## Abstract

Southern tick-associated rash illness (STARI) is an emerging zoonotic disease causing an annular rash with central clearing that is almost identical to erythema migrans seen in Lyme disease. It is spread by *Amblyomma americanum* tick bite. Although it is still debatable, this zoonotic disease is thought to be caused by *Borrelia lonestari* spirochete. At this time, there is no approved diagnostic modality nor approved treatment for such an illness. Here we describe a rare case of STARI in a 63-year-old female and shed light on the differences between STARI and Lyme disease.

## Introduction

Southern tick-associated rash illness (STARI) or Master’s disease is an emerging Lyme-like illness in the southeastern and south-central United States. It is vectored by the lone-star tick *Amblyoma americanum*. Although it is still debatable, the disease is thought to be caused by *Borrelia lonestari* spirochete based on isolating the bacterium in a single case of STARI [1]. The associated rash is similar if not indistinguishable from Lyme disease erythema migrans, with lymphocytic dermal infiltrate. Here we present a case of STARI with pancytopenia to help in raising the clinical awareness and knowledge of Lyme disease mimic.

## Case presentation

**Figure 1:**
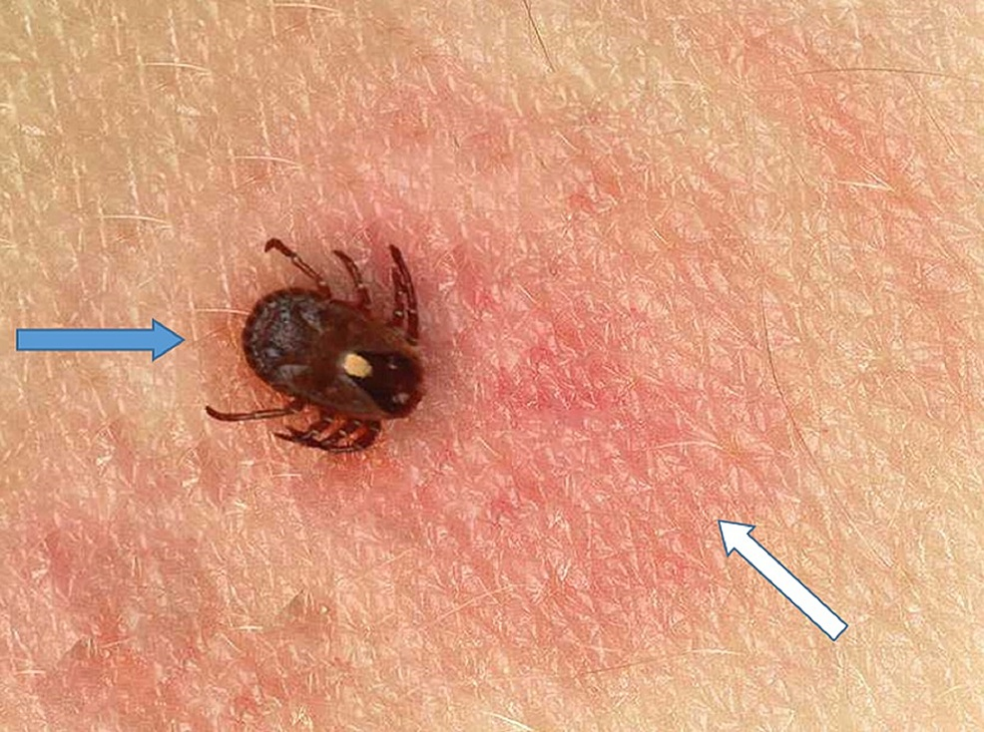
The isolated lone star tick (blue arrow) causing Southern tick-associated rash illness, and the target erythematous lesion on the right leg (white arrow) at the time of tick isolation

**Figure 2:**
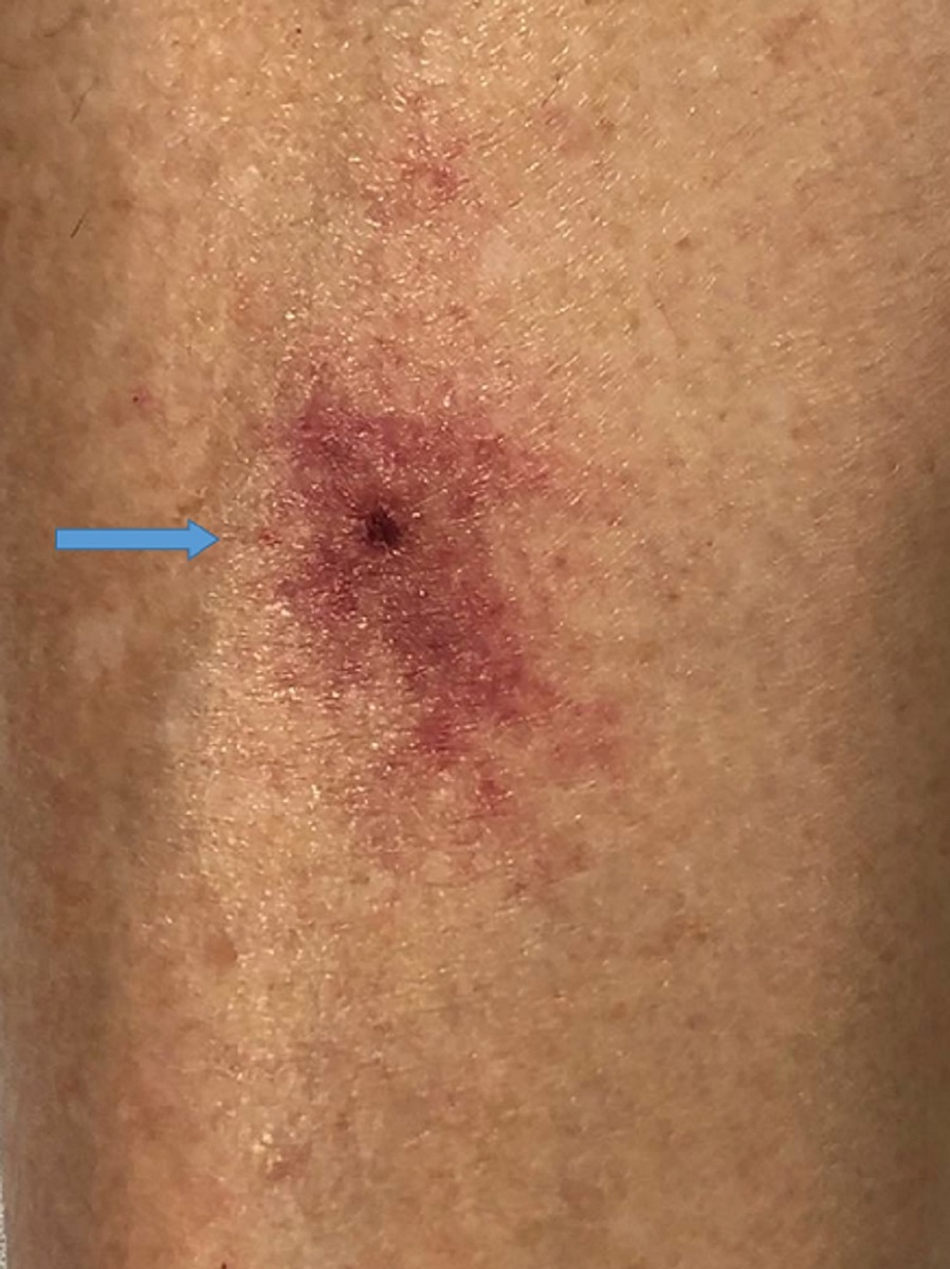
Target erythematous lesion on the right leg (blue arrow) at the time of presentation

A 63-year-old female with unremarkable past medical history presented with persistent fever, headache, and diffuse myalgia for four days after returning from a camping trip in Gainesville, Florida two weeks prior. She also reported noticing a tick on her right leg (Figure 1) and having a pruritic target erythematous lesion after removing it. On presentation, she was febrile at 100.5 F and tachycardic at 127 bpm. She had an erythematous ecchymotic area on her anterior right shin surrounding the location of the reported tick bite (Figure 2).

**Figure 3:**
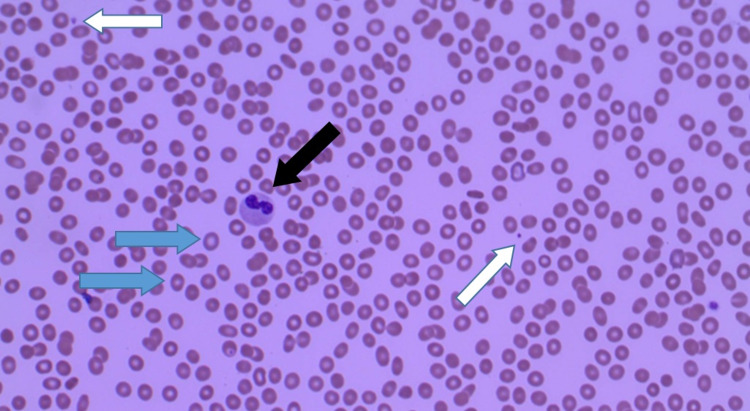
Peripheral blood smear at the time of presentation showing pancytopenia. Low red blood cells count (blue arrow), low white blood cells count (black arrow), and low platelets count (white arrow)

Her laboratory workup was remarkable for pancytopenia with white blood cells (WBC) 1.1k/mm^3^, neutrophils 0.4k/mcL, red blood cells (RBC) 3.23 million/mcL, and platelets 27k/mm^3^. Aspartate transaminase was 316 units/L and alanine transaminase 169 units/L. Her chest radiograph and urine analysis were negative. Ehrlichiosis, anaplasmosis, Lyme disease, and hepatitis panels were negative. Peripheral smear showed pancytopenia without inclusion bodies or morula (Figure 3). The patient was started on doxycycline 100 mg twice a day for 14 days. On day 5, she reported improvement of symptoms. Her pancytopenia resolved with WBC 6.7k/mm^3^, neutrophils 2.2k/mcL, RBC 3.48 million/mcL, and platelets 151k/mm^3^. Her rash resolved after one month.

## Discussion

STARI is an emerging tick-borne zoonotic disease that has been an enigma in non-endemic states for the past 30 years. STARI meets the clinical and surveillance criteria of Lyme disease but not the microbiologic definition [2]. The causative organism remains controversial, as *Borrelia lonestari* is not always isolated in STARI cases [3], questioning whether there are other possible etiologies. The STARI rash is almost similar to Lyme disease erythema migrans. It tends to be smaller, more circular with central clearing, less uniform in color and pattern, and less tender. Patients with STARI can develop non-specific symptoms of fever, malaise, and body aches, but less likely to have neck stiffness, arthralgia, and regional lymphadenopathy. No long-term sequelae have been reported.

At the present time, there is no approved diagnostic modality to identify STARI; thus, the diagnosis must be made on clinical evidence including erythema migrans and tick exposure. Distinguishing STARI from Lyme disease is very complex due to their tremendous overlaps and similarities. Diagnosis usually relies on geographic association (STARI from central Texas and Oklahoma eastward across the southern states and along the Atlantic coast as far north as Maine, versus Lyme disease in northeast, mid-Atlantic, and upper mid-west), clinical presentation, laboratory workup (Lyme disease serologic tests), and long-term sequelae [4]. STARI is often treated as Lyme disease with doxycycline twice daily for 14 days; however, there is no approved treatment yet [5]. Physicians should be aware of this emerging zoonosis and its acute presentation. Further research into the prevalence, causative organisms, laboratory testing, and proper treatment of STARI is warranted.

## Conclusions

STARI is an emerging Lyme-like illness that causes the characteristic rash, erythema migrans. The current incidence of STARI remains unknown as it is not nationally reportable. A recent study has suggested that STARI is transmitted by the lone-star tick *Amblyoma americanum*; however, it may take some time before all the necessary data can be collected, since much is still unknown about STARI. Patients suspected of having STARI seem to respond well to doxycycline.

## References

[1] Varela AS, Luttrell MP, Howerth EW, Moore VA, Davidson WR, Stallknecht DE, Little SE (2004). First culture isolation of Borrelia lonestari, putative agent of southern tick-associated rash illness. J Clin Microbiol.

[2] Blanton L, Keith B, Brzezinski W (2008). Southern tick-associated rash illness: erythema migrans is not always Lyme disease. South Med J.

[3] Kannangara DW, Patel P (2018). Report of non-Lyme, erythema migrans rashes from New Jersey with a review of possible role of tick salivary toxins. Vector Borne Zoonotic Dis.

[4] Goldstein IM, Black MA, Leydet B Jr, Vidrine SB (2013). Hitting the target: Lyme or STARI?. J La State Med Soc.

[5] Molins CR, Ashton LV, Wormser GP (2017). Metabolic differentiation of early Lyme disease from southern tick-associated rash illness (STARI). Sci Transl Med.

